# Genetic Variants in the Insulin-like Growth Factor Pathway and Colorectal Cancer Risk in the Netherlands Cohort Study

**DOI:** 10.1038/srep14126

**Published:** 2015-09-18

**Authors:** Colinda C. J. M. Simons, Leo J. Schouten, Roger W. L. Godschalk, Manon van Engeland, Piet A. van den Brandt, Frederik J. van Schooten, Matty P. Weijenberg

**Affiliations:** 1 Department of Epidemiology, GROW – School for Oncology and Developmental Biology, Maastricht University, Maastricht, the Netherlands; 2 Department of Toxicology, NUTRIM – School of Nutrition and Translational Research in Metabolism, Maastricht University, Maastricht, the Netherlands; 3 Department of Pathology, GROW – School for Oncology and Developmental Biology, Maastricht University Medical Centre, Maastricht, the Netherlands

## Abstract

Interrelationships between insulin-like growth factors (IGFs), hyperinsulinaemia, diabetes, and colorectal cancer (CRC) indicate involvement of IGFs in colorectal tumorigenesis. We investigated the CRC risk associated with 24 single nucleotide polymorphisms (SNPs) in 9 genes related to the IGF pathway and an *IGF1* 19-CA repeat polymorphism. Variants were selected from literature and genotyped in toenail DNA from 3,768 subcohort members and 2,580 CRC cases from the Netherlands Cohort Study, which has a case-cohort design (n = 120,852). We used the follow-up period 1986–2002. Eighteen SNPs were unequivocally associated with selected endpoints in the literature and unfavorable alleles were aggregated into a genetic sum score. Cox regression showed that a higher genetic sum score significantly increased CRC risk at all subsites, except the rectum, in men (highest vs. lowest tertile: HR for CRC = 1.36, 95% CI: 1.11, 1.65; *P*-trend = 0.002). Single SNPs (except the *IGF1* SNP rs5742694) were not associated with risk. Models including the total number of *IGF1* 19-CA repeats showed CRC risk was halved at all subsites in women carrying <38 repeats but not >38 repeats (≤36 versus 38 repeats: HR for CRC = 0.44; 95% CI: 0.33, 0.58; *P*-trend < 0.001). These findings support a role for variants in IGF-related genes in colorectal tumorigenesis.

The insulin-like growth factor (IGF) pathway is involved in normal growth and putatively colorectal tumorigenesis. In support of this, blood levels of IGF-related factors have been associated with CRC risk^1^. In addition, an increased IGF-1 level, the main growth factor in adult life, has been associated with hyperinsulinaemia, which may result in insulin resistance and, ultimately, type 2 diabetes mellitus^2^. Type 2 diabetics have been shown to be at an increased risk of CRC^3^. Furthermore, since hyperinsulinaemia can stimulate the production of IGF-1, adiponectin and peroxisome proliferator-activated receptor gamma may be considered, as these factors have been associated with glucose and lipid homeostasis, insulin resistance, and compensatory hyperinsulinaemia^2^,^4^,^5^.

A genetic predisposition to CRC regarding IGF-related factors would substantiate a role for the IGF pathway in colorectal tumorigenesis. Indeed, genetic variants in genes encoding for IGF-related factors have been associated with CRC risk in several studies^6^,^7^,^8^,^9^,^10^,^11^,^12^,^13^,^14^,^15^,^16^,^17^,^18^,^19^,^20^,^21^,^22^,^23^,^24^,^25^,^26^,^27^,^28^, but integration of genetic information from across genes in the IGF pathway is lacking. This is important, because most single SNPs confer only minor risks and because gene-gene(-environment) interactions^29^ and functional compensation between genes may exist^30^. Therefore, we set out to add to the existing molecular epidemiological evidence by using a genetic sum score of unfavorable alleles, which also allows quantifying sex- and subsite-specific CRC risks with optimal power. Quantifying sex- and subsite-specific risks is important, because CRC is a heterogeneous disease, with risk factors differing between men and women and for different anatomical subsites^31^.

Previous studies have shown genetic sum scores to be successful for investigating multiple SNPs at once in relation to carcinogenic biomarkers [e.g.^32^,^33^,^34^], cancer risk [e.g.^35^,^36^,^37^,^38^,^39^], and cancer survival [e.g.^40^,^41^,^42^,^43^]. As opposed to many of these studies, we used the literature to define unfavorable alleles in terms of those potentially increasing CRC risk. We required that SNPs had been significantly associated with a selected endpoint at least twice or with >1 selected endpoints in the literature (an exception was made for missense variants). Unless SNPs were equivocally associated with selected endpoints across studies, unfavorable alleles were aggregated into a genetic sum score. Our literature-based strategy avoided overfitting of the cumulative model due to potential false-positive findings in a single dataset. We conservatively refrained from weighing SNPs in the score according to their associated effect size, because the effect of a single SNP is likely dependent on other SNPs and environmental factors [gene-gene(-environment) interactions].

Our hypothesis was that carrying more unfavorable alleles in genes related to the IGF pathway, as indicated by a higher genetic sum score, increases CRC risk. Our study population was the Netherlands Cohort Study, which includes 120,852 men and women. In addition to SNPs, we also studied an *IGF1* 19-CA repeat polymorphism that has been associated with CRC risk in several studies, though not consistently^7^,^9^,^10^,^13^,^23^,^24^,^25^,^28^.

## Results

### Baseline characteristics

Genotype and allele frequencies, the *P*-value for Hardy-Weinberg Equilibrium (HWE), and the unfavorable allele as indicated by literature are shown in Table 1. All SNPs adhered to HWE in subcohort members, except SNP rs1342387. We did not exclude SNP rs1342387, because all SNPs were genotyped simultaneously and there was no indication of genotyping errors. Unfavorable alleles for 18 SNPs in genes related to the IGF pathway were aggregated into a genetic sum score, as the literature was unequivocal about the direction of the association for these SNPs. Tertiles of the genetic sum score ranged from 6–14, 15–18, and 19–29 unfavorable alleles. The theoretical maximum was 36. 134 subcohort members and 120 CRC cases could not be categorized into one of the tertiles because of missing SNP data (one SNP was missing at most; this was the case in a total of 356 subcohort members and 311 CRC cases). The *IGF1* 19-CA repeat, for which we distinguished between individuals carrying 19/19, 19/non-19, and non-19/non-19 CA repeats, was not in HWE in the subcohort, when taking into account the multiallelic character of this locus (P-value < 0.001), although deviations may arise due to the presence of rare alleles and genotypes, which is the case in our population.

Lifestyle characteristics and dietary habits in men and women according to tertiles of the genetic sum score are shown in Table 2. Men in different tertiles of the genetic sum score did not differ on any of the baseline characteristics. Women in higher tertiles of the genetic score were more likely to drink more alcohol, consume more meat and have a higher total energy intake (*P*-values < 0.05); there were no differences in age, family history of CRC, diabetes after age 30, anthropometric measures, physical activity levels, smoking status, and meat intake.

### Genetic sum score

Table 3 shows the associations of the genetic sum score in tertiles with CRC risk by subsite in men and women as derived from age-adjusted Cox models. The findings in Table 3 show that an accumulation of unfavorable alleles in the IGF pathway may increase CRC risk in men but not women. Specifically, we observed dose-response relationships between the genetic sum score and CRC risk at all subsites, except the rectum, in men. Men in the highest versus lowest tertile were at an ~40% increased risk [hazard ratio (HR) for CRC = 1.36, 95% CI: 1.11, 1.65, *P*-trend = 0.002; HR for colon cancer = 1.39, 95% CI: 1.11, 1.74, *P*-trend = 0.004; HR for proximal colon cancer = 1.34, 95% CI: 1.00, 1.79, *P*-trend = 0.06; HR for distal colon cancer = 1.48, 95% CI: 1.12, 1.94, *P*-trend = 0.006]. The genetic sum score was not associated with CRC risk by subsite in women, although a trend towards an increased rectal cancer risk was observed (middle and highest versus lowest tertile: HR = 1.28, 95% CI: 0.85, 1.91 and HR = 1.50, 95% CI: 0.98, 2.28, respectively; *P*-trend = 0.06). Models, in which we estimated the risks associated with the number of unfavorable alleles as a continuous variable, showed hazard ratios to be significantly increased with 3–4% for each additional unfavorable allele at all CRC subsites, except the rectum, in men but not women. For interpretability, this translates into 30–40% increased CRC risks per 10 additional unfavorable alleles under the assumption of linearity.

Single SNPs were not associated with CRC risk, except the *IGF1* SNP rs5742694 in men (Table 4). Considering this finding and that *IGF1* is central in the IGF pathway, we examined whether *IGF1* SNPs were drivers of the associations observed with respect to the genetic sum score. We modeled a genetic sum score that did not include *IGF1* SNPs. The model was adjusted for the excluded variants to make sure effects were independent of these SNPs. This simultaneously provided a check as to whether the LD between SNPs in the *IGF1* gene influenced results, as there was higher LD between these SNPs (median: 0.650; range: 0.327–0.872) than between the other SNPs (median: 0.306; range: 0.090–0.753). Results showed no essential differences (data not shown).

Models including separate genetic sum scores for 1) SNPs in genes encoding for factors in or regulatory to the IGF pathway and 2) SNPs in genes encoding for adiponectin, adiponectin receptors, and the peroxisome proliferator-activated receptor gamma, did not yield essentially different results as compared to results from models including the overall genetic sum score (data not shown). Additional adjustment for the number of SNPs and genes, respectively, underlying the genetic sum score attenuated the previously increased proximal colon cancer risk in men, rendering this risk nonsignificant (data not shown). Only when we adjusted for the number of SNPs underlying the genetic sum score did we observe changes in results in women, *i.e*. we observed a significantly increased colon cancer risk, particularly for the distal colon (data not shown). When we modelled the number of genes with unfavorable alleles in a continuous fashion, we observed a 42–48% increased CRC risk at all subsites in both men and women (Supplementary Table 2).

### IGF1 19-CA repeat

Table 3 also shows the associations of the *IGF1* 19-CA repeat polymorphism using the categorization by Rosen *et al*.^44^ and using a sum of repeats on both chromosomes with CRC risk by subsite in men and women as derived from age-adjusted Cox models. The findings in Table 3 show that variant repeat alleles may decrease CRC risk in women but not men. Specifically, using the categorization by Rosen *et al*.^44^, the *IGF1* 19-CA repeat was not associated with CRC risk in men. In women, there was evidence of dose-response relationships with CRC risk at all subsites, except the rectum. CRC risk was about halved in women homozygous for variant (non-19-CA) repeat alleles versus women homozygous for the wild type (19-CA) repeat allele (HR for CRC = 0.54, 95% CI: 0.42, 0.70, *P*-trend <0.001; HR for colon cancer = 0.50, 95% CI: 0.38, 0.65, *P*-trend < 0.001; HR for proximal colon cancer = 0.48, 95% CI: 0.34, 0.67, *P*-trend < 0.001 and HR for distal colon cancer = 0.52, 95% CI: 0.36, 0.75, *P*-trend = 0.001). A model including the sum of repeats on both chromosomes, using individuals carrying 38 CA repeats as the reference group, revealed that risk reductions, including a reduced rectal cancer risk, were present in women carrying less than 38 CA repeats, but not more than 38 CA repeats. Exclusion of individuals not homozygous for the wild type allele (19 CA repeats) from the reference category, slightly strengthened associations (data not shown).

## Discussion

Current data showed that an accumulation of unfavorable alleles with respect to SNPs in genes related to the IGF pathway increased CRC risk at all subsites, except the rectum, in men. Single SNPs (except one) were not associated with CRC risk, underlining the importance of integrating SNP information across genes in a pathway. This study builds on a number of previous studies on SNPs in genes related to the IGF pathway and CRC risk^6^,^7^,^8^,^9^,^10^,^11^,^12^,^13^,^14^,^15^,^16^,^17^,^18^,^19^,^20^,^21^,^22^,^23^,^24^,^25^,^27^,^28^ and provides further evidence for the involvement of the IGF pathway in colorectal tumorigenesis. Findings between studies are difficult to compare due to that different sets of SNPs and mostly single SNP effects were studied. However, few studies have shown such clear dose-response relationships as the present one.

With respect to the *IGF1* 19-CA repeat polymorphism, we observed a reduced CRC risk to be associated with variant alleles in women when using the classical categorization by Rosen *et al*.^44^ A risk reduction at all subsites was present in women with less than 38 repeats but not in women with more than 38 repeats on both chromosomes together when we distinguished between individuals carrying less or more than 38 repeats. This is the most common total number of repeats, which in the majority of individuals corresponds to being homozygous for the wild type allele. We hypothesized in our methods section that the number of repeats may influence CRC risk differently. The categorization of individuals with fewer and more than 19 CA repeats (the wild type allele) in the same category—as is done in the classical categorization—may have yielded both increased and decreased risks in previous studies for variant alleles depending on the distribution of the number of repeats in a particular study population^9^,^23^,^24^,^25^. Our results support our hypothesis that the number of repeats may matter and future studies are encouraged to model the total number of *IGF1* 19-CA repeats to elucidate this further.

Next, some methodological considerations associated with the use of a genetic sum score should be made. First, an advantage of a genetic sum score is that no explicit assumption on the inheritance mode of the SNPs is necessary (recessive/dominant/additive). That is, if one treats all SNPs according to an additive inheritance mode and one assumes that one is not more likely to include SNPs adhering to a recessive than a dominant inheritance mode, the misclassification associated with assuming an additive inheritance mode is likely to cancel out in the sum score. To explain this, consider the example of a genetic sum score for two SNPs, of which one SNP adheres to a recessive inheritance mode and the other adheres to a dominant inheritance mode. For both SNPs, we assume an additive inheritance mode. If an individual is heterozygous for both SNPs and we aggregate the number of unfavorable alleles, we arrive at a sum score of 2. Had we known that one SNP adhered to a recessive inheritance mode, meaning that one unfavorable allele does not influence CRC risk, we should have coded the heterozygous genotype the same as the homozygous genotype for the other allele, *i.e*. ‘0’ (instead of ‘1’). Had we known that the second SNP adhered to a dominant inheritance mode, meaning that carrying one or two unfavorable allele(s) influences CRC risk in the same way, we should have coded the heterozygous genotype the same as the homozygous genotype for the unfavorable allele, *i.e*. ‘2’ (instead of ‘1’). If we aggregate these two scores, we arrive at a sum score of 2, which is the same score as the score arrived at when assuming an additive inheritance mode.

Second, a particular genetic sum score may influence CRC risk differently depending on the number of SNPs and genes, respectively, underlying the sum. For example, a score of 10 can be achieved by being heterozygous for 10 SNPs or by being homozygous for the unfavorable allele for five SNPs (or any combination in between). Likewise, a sum of 10 can correspond to carrying unfavorable alleles in five, six, seven, eight, nine, or ten different genes. Both types of adjustment in our dataset influenced results, indicating that the number of SNPs and genes underlying the genetic sum score may be relevant weighing factors. Furthermore, our finding that the number of genes with unfavorable alleles by itself increased CRC risk at all subsites with 42-48% in both men and women, suggests that an accumulation of unfavorable alleles *across genes* in a pathway may be particularly important for influencing CRC risk.

Third, as mentioned in the introduction, SNP effects may differ, which is why the effect size for individual SNPs has been used as a weight in genetic sum scores. As explained, we refrained from doing so, because the strength of single SNP effects may depend on other SNPs and environmental factors. Our approach was conservative, because it remains to be seen whether a better estimation of risk is achieved when weighing a genetic sum score according to single SNP effects and/or the number of SNPs or genes underlying the score as suggested in the previous paragraph. This should ideally be investigated using simulated data in which true SNP effects and interaction patterns are known. However, it is important to realize that our conservative approach, in which SNPs are not weighed, only holds under the assumption that single SNPs have similar effects on risk. This may be a reasonable assumption in our data, considering that all included SNPs were common SNPs (MAF > 5%), which may be hypothesized to have modest effects on common cancers like CRC^45^. As mentioned in the methods section, we did not include the *IGF1* 19-CA repeat polymorphism, because it is a different type of variant than a SNP for which this assumption is less likely. Still, misclassification of individuals on the genetic sum score can never be excluded, and since this misclassification was likely independent of disease, it most probably attenuated results.

Finally, it may matter whether or not unfavorable alleles within a gene were located on the same parental chromosome. This predominantly applies to individuals who carry heterozygous genotypes. We were unable to explore a potential influence on risk of this, because we could not determine the chromosomal origin (paternal or maternal) of alleles.

Despite these considerations, the use of a genetic sum score for SNPs was successful in this study. Strengths of this study include the prospective design and long follow-up with large case numbers. A limitation may be that only ~75% of the cohort returned toenail clippings. However, comparison of subcohort members with and without available toenail material on baseline characteristics did not indicate this to be a selective group (data not shown).

Future directions include investigating whether an accumulation of unfavorable alleles in genes related to the IGF pathway modifies the increased CRC risk associated with overweight and a lack of physical activity. Overweight and physical activity has been associated with blood levels of IGF-related factors^1^, and are likely candidates to interact with genetic variants in genes related to the IGF pathway. Previous gene-environment interaction (GxE) studies on this, however, have been inconsistent^25^,^46^,^47^. In future GxE studies, the use of a genetic sum score will be advantageous, particularly its associated optimization in power, because the detection of a statistical significant interaction has been estimated to require a four times larger sample size as compared to the detection of a marginal effect of similar magnitude^48^. The results of GxE studies can contribute to the evidence base underlying the development of targeted CRC prevention strategies aimed at modifying diet and lifestyle. Genetic sum scores, in this regard, might be useful variables for risk stratification.

In conclusion, an accumulation of unfavorable alleles increased CRC risk in men, whereas a decreased total number of *IGF1* 19-CA repeats reduced CRC risk in women. These findings provide further evidence for the involvement of the IGF pathway in colorectal tumorigenesis. That single SNPs were not associated with CRC risk underlines the importance of integrating SNP information across genes in a pathway.

## Materials and Methods

### Study population and design

The Netherlands Cohort Study (NLCS) includes 120,852 men and women who were between 55–69 years old in 1986, when completing a self-administered baseline questionnaire on diet and cancer. Participants originate from the general population in the Netherlands and were sampled via the municipal population registries. The NLCS has been described in detail previously^49^. Along with the questionnaire, participants were asked to return toenail clippings by way of an enclosed envelope. ~90,000 participants provided toenail clippings, which is a valid DNA source for the genotyping of germline genetic variants^50^. Toenail DNA isolation is performed according to the DNA extraction protocol of Cline *et al*.^51^ with some adjustments^50^. The NLCS, the use of toenail DNA for genotyping, and associated protocols were approved by the review boards of the TNO Nutrition and Food Research Institute (Zeist, the Netherlands) and Maastricht University (Maastricht, the Netherlands). All methods were carried out in accordance with the approved guidelines.

The NLCS is characterized by a case-cohort design, which entails that a random subcohort (n = 5,000), selected immediately after baseline, is followed up to estimate the accumulated person-time at risk, whereas incident cancer cases are enumerated for the entire cohort. Follow-up for vital status is performed through linkage to the Central Bureau of Genealogy and the municipal population registries (~100% completeness). Cancer follow-up is performed through linkage with the population-based cancer registry and PALGA (the Netherlands pathology database) (>96% completeness)^52^,^53^. After 16.3 years and exclusion of participants with a history of cancer other than skin cancer at baseline, there were 4,774 subcohort members and 3,440 incident CRC cases. Toenail DNA was available for 3,768 of these subcohort members (78.9%) and 2,580 of these CRC cases (75.0%); 114 subcohort members who developed CRC during follow-up were included in both counts, leaving a total of 6,234 unique toenail DNA samples.

### Gene and SNP selection

Our gene and SNP selection strategy was literature-based and ultimately intended for studying GxE interactions between genetic variants in genes related to the IGF pathway and body size, physical activity, and energy restriction. We selected genes in or regulatory to the IGF pathway (*i.e. IGF1*, *IGF2*, *IGF1R*, *IGF2R*, *IRS1*, *IRS2*, *IGFBP1-7*, *IGFALS*, *GH1*, *GHR*, *GHRH*, and *GHRHR*), genes related to adiponectin (*i.e. ADIPOQ*, *ADIPOR1*, *ADIPOR2*), and the peroxisome proliferator-activated receptor gamma gene (*PPARG*). We accepted that our literature-based SNP selection strategy may neglect false negative findings in the literature as we primarily aimed at replicating previous findings and quantifying sex- and subsite-specific CRC risks through the use of a genetic sum score.

Our SNP selection strategy consisted of four steps. In step 1, we searched Pubmed for studies on SNPs in the selected genes in relation to the following endpoints: i) the risk of CRC; ii) traits of interest in the context of future GxE work, *i.e*. obesity, insulin resistance, and blood levels of IGF pathway-related factors; iii) the risk of type 2 diabetes mellitus; and iv) the risk of other obesity-related cancers. We also searched for hits in these genes in genome-wide association studies on v) CRC, vi) type 2 diabetes mellitus, and vii) traits of interest as described under ii). The search yielded 381 SNPs with a reported rs-number. We carried forward SNPs with a >10% prevalence of the rare homozygous and heterozygous genotypes as reported in CEU individuals from the Hapmap project to step 2 (n = 275).

In step 2, SNPs were scored on points i through vii. If no association was reported in relation to a specific endpoint, SNPs were assigned a selection score of ‘0’ for that endpoint; if found associated once, SNPs were assigned a selection score of ‘1’; if found associated at least twice, SNPs were assigned a selection score of ‘2’. We chose not to assign selection scores of ‘3’ or higher when SNPs were found associated with a specific endpoint in more than two studies, because this might simply lead to the prioritization of SNPs that have often been investigated (see step 3).

In step 3, selection scores across points i–vii were summed and used to rank SNPs. In order to minimize the chance of selecting SNPs based on false-positive results, SNPs with a sum score of less than two were excluded (n = 222), with the exception of four missense SNPs. One SNP of the remaining 53 SNPs was in perfect LD with another SNP (r^2^ = 1). A second SNP failed in a pilot that preceded this study and could not be replaced with a perfect proxy (r^2^ = 1). These two SNPs were therefore excluded.

Step 4 concerned the assay design using the iPLEX^TM^ assay for genotyping on the SEQUENOM^®^ MassARRAY^®^ platform (Sequenom Inc., Hamburg, Germany). This platform allows high-throughput genotyping of maximally 40 SNPs at once. Considering that multiplexing is often not 100% efficient due to sequence incompatibilities between the sequences flanking the SNPs, we designated the 20 highest ranked SNPs as high-priority SNPs (all had a total selection score of ≥3). The remaining 31 low-priority SNPs were used for ‘superplexing’, *i.e*. given a fixed, optimal design as based on the set of high-priority SNPs, these SNPs were added to the design if possible. In total, 25 SNPs in 9 genes could be included in the assay, of which 15 high-priority and 10 low-priority SNPs.

### SNP genotyping

The protocol for genotyping on the SEQUENOM^®^ MassARRAY^®^ platform has been described previously^54^ and was carried out using 100 ng of toenail DNA, pipetted into 384-well plates. Included were duplicate samples for a random selection of 314 samples and 436 water controls. Twenty-four out of the 25 SNPs in the assay were successfully genotyped. Genotyping of SNP rs35767 failed as only the C-allele was found. The reproducibility of genotypes was 98.8% or higher for the different SNPs. Exclusion of possibly contaminated samples as indicated by our laboratory technicians (n = 4), samples with irreproducible results (n = 1), and samples with a call rate <95% (n = 532, 8.5%) resulted in 5,697 samples. All SNPs had call rates of 92.6% or higher, except SNP rs4773082, which had a call rate of 83.6%.

### Genotyping of the IGF1 19-CA repeat polymorphism

The *IGF1* 19-CA repeat polymorphism was genotyped by PCR amplification and subsequent analysis of the PCR products’ length using the 96-capillary ABI 3730xl DNA Analyzer. The PCR was carried out using 100 ng of DNA, 10.75 μl MilliQ, 2.5 μl 10x PCR buffer, 0.875 μl of 50 mM MgCL2, 2 × 0.125 μl of Primer predilution-mix (10 times diluted), 0.5 μl of 10 mM dNTP mix, and 0.125 μl of Platinum Taq Polymerase (Life Technologies, Bleiswijk, the Netherlands). The primers (forward: 5′-ACCACTCTGGGAGAAGGGTA-3′; reverse: 5′-GCTAGCCAGCTGGTGTTATT-3′) were fluorescently labelled with 6-FAM (blue), NED (yellow), and PET (red), which enabled the simultaneous analysis of three samples in a single run on the ABI 3730xl DNA Analyzer. The protocol was carried out in the dark because of the light-sensitivity of the fluorescent labels. The PCR reactions were performed using the following cycles: 94 °C for 10 min, followed by 35 cycles of 94 °C for 30 sec, 55 °C for 30 sec, and 72 °C for 30 sec, followed by 72 °C for 10 min and 4 °C for 30 min. The analysis included 314 duplicate samples and 436 water controls. The reproducibility of the *IGF1* 19-CA repeat analysis was 93.6%. Genotyping was successful for 70.7% of samples.

### Statistical analysis

Hazard ratios and 95% confidence intervals were estimated using age-adjusted Cox regression models. We conservatively refrained from including other CRC risk factors as covariates in the model, particularly indicators of body size which may have a genetic basis associated with the IGF pathway, because adjusting for covariates with a potential genetic basis may unintentionally introduce (collider) bias in genetic association studies^55^. Participants with inconsistent/incomplete baseline questionnaires were excluded in order to relate genotyping results to baseline characteristics and to keep numbers comparable with those in future GxE studies within the NLCS. This left 3,203 subcohort members and 2,274 CRC cases with SNP data, and 2,134 subcohort members and 1,833 CRC cases with data on the *IGF1* 19-CA-repeat. To calculate a genetic sum score, we aggregated unfavorable alleles for SNPs, unless the literature was equivocal about the direction of the association with selected endpoints. Alleles were considered ‘unfavorable’ if these increased the risk of selected diseases [CRC, type 2 diabetes mellitus, and other obesity-related cancers (i.e. cancers of the oesophagus, pancreas, gallbladder, breast (in postmenopausal women), endometrium, and kidney^56^)], or if these were associated with selected traits in a manner that may increase CRC risk (overweight, obesity, insulin resistance, and blood levels of IGF pathway-related factors). In a two-SNP example, an individual heterozygous for one SNP and homozygous for the unfavorable allele on the other SNP would receive a sum score of 1 + 2 = 3. The genetic sum score was categorized into tertiles as based on the distribution in the subcohort. Tertiles enabled tests for a linear trend, while maintaining optimal power within categories. Categorization of the genetic sum score allows for better interpretability of the results, considering that the human genome consists of millions of SNPs and considering that our inclusion of potentially relevant SNPs was not exhaustive. However, for completeness, we also modelled the genetic sum score in a continuous fashion. SNPs not included in the genetic sum score were evaluated when analyzing single SNPs.

Single SNPs were analyzed assuming an additive inheritance mode and only in relation to the overall CRC risk in men and women, because of power considerations. We furthermore conducted four sensitivity analyses. First, we modeled a sum score only including SNPs in genes encoding for factors in or regulatory to the IGF pathway and a sum score only including SNPs in genes encoding for adiponectin, adiponectin receptors, and peroxisome proliferator-activated receptor gamma, because these factors may be conceptually different. Second and third, we additionally adjusted our model that included the genetic sum score for the number of SNPs and genes, respectively, underlying an individual’s score; these variables might turn out relevant weighing factors. Fourth, we modeled the number of genes in which unfavorable alleles were present to explore whether an accumulation of unfavorable alleles *across genes* may be important for influencing risk.

The *IGF1* 19-CA repeat polymorphism was categorized according to Rosen *et al*.^44^, distinguishing individuals homozygous for the wild type allele (19/19 CA repeats), heterozygous individuals (19/non-19 CA repeats), and individuals homozygous for variant alleles (non-19/non-19 CA repeats). We did not include the *IGF1* 19-CA repeat polymorphism in the genetic sum score because it is a conceptually different type of variant than a SNP. This means that the assumption that all variants in the genetic sum score have a similar weight is less assured for this variant. In light of that previous studies showed increased^9^,^24^,^25^ and decreased CRC risks^23^ for variant alleles, we hypothesized that the number of repeats may influence CRC risk differently: *i.e*., categorizing individuals with fewer and more than 19 CA repeats (the wild type allele) in the same category may have led to qualitatively different observations depending on the distribution of the variant alleles in a particular study population. To explore this, we considered a model in which the number of repeats on both chromosomes was aggregated. Essentially, this yielded another sum score which was analyzed categorically. Individuals with 38 repeats—most of which were homozygous for the wild type allele (19 CA repeats)—were taken as the reference group.

In all analyses, standard errors were estimated using the robust Huber-White sandwich estimator to account for the additional variance introduced by sampling the subcohort from the entire cohort. The proportional hazards assumption was tested using the scaled Schoenfeld residuals and by visually inspecting the -log-log-transformed hazard curves (there were no apparent violations). All analyses were conducted using Stata version 12 (Stata Corp., College Station, TX). Statistical significance was indicated by a *P*-value < 0.05 for two-sided testing. We did not correct for multiple testing because our study was hypothesis-based and because our use of a genetic sum score of unfavorable alleles (the primary mode of analysis) greatly reduced the number of tests that had to be performed.

## Additional Information

**How to cite this article**: Simons Colinda, C.J.M. *et al*. Genetic Variants in the Insulin-like Growth Factor Pathway and Colorectal Cancer Risk in the Netherlands Cohort Study. *Sci. Rep*. **5**, 14126; doi: 10.1038/srep14126 (2015).

## Supplementary Material

Supplementary Information

## Figures and Tables

**Table 1:** Minor allele and genotype frequencies of single nucleotide polymorphisms in genes related to the IGF pathway in subcohort members from the Netherlands Cohort Study (1986–2002)^a^.

Variant	Gene	Genelocation	Majorallele	Minorallele	MAF	Homozygouscommon genotypeN (%)	Heterozygousgenotype N (%)	Homozygous raregenotype N (%)	*P* forHWE	Unfavorable alleleas based onliterature^b^
rs1520220	*IGF1*	12q23.2	C	G	0.18	2,292 (67.6)	989 (29.2)	110 (3.2)	0.79	Minor
rs5742678	*IGF1*	12q23.2	C	G	0.25	1,921 (56.7)	1,250 (36.9)	220 (6.5)	0.39	Minor
rs5742694	*IGF1*	12q23.2	T	G	0.23	2,019 (59.5)	1,172 (34.6)	200 (5.9)	0.09	Minor
rs10735380	*IGF1*	12q23.2	A	G	0.26	1,886 (55.6)	1,271 (37.5)	234 (6.9)	0.32	Minor
rs2854744	*IGFBP3*	7p13-p12	C	A	0.45	1,002 (29.6)	1,695 (50.0)	693 (20.4)	0.63	Major
rs2132572	*IGFBP3*	7p13-p12	G	A	0.23	2,006 (59.2)	1,198 (35.3)	187 (5.5)	0.64	Minor
rs35440925	*IGFBP3*	7p13-p12	C	G	0.21	2,110 (62.2)	1,137 (33.5)	144 (4.3)	0.55	Unclear
rs1801278	*IRS1*	2q36	G	A	0.07	2,926 (86.3)	450 (13.3)	15 (0.4)	0.60	Minor
rs1805097	*IRS2*	13q34	G	A	0.35	1,431 (42.4)	1,530 (45.3)	416 (12.3)	0.82	Major
rs2289046	*IRS2*	13q34	A	G	0.34	1,466 (43.2)	1,538 (45.4)	387 (11.4)	0.59	Major
rs754204	*IRS2*	13q34	C	T	0.48	906 (27.2)	1,689 (50.6)	740 (22.2)	0.37	Minor
rs4773082	*IRS2*	13q34	T	C	0.43	994 (32.2)	1,518 (49.1)	578 (18.7)	0.97	Minor
rs4988496	*GHRHR*	7p14	G	A	0.06	3,008 (88.7)	371 (10.9)	11 (0.3)	0.90	Minor
rs1801282	*PPARG*	3p25	C	G	0.12	2,607 (76.9)	739 (21.8)	43 (1.3)	0.25	Major
rs1501299	*ADIPOQ*	3q27	C	A	0.26	1,852 (54.6)	1,290 (38.0)	249 (7.3)	0.24	Major
rs2241766	*ADIPOQ*	3q27	T	G	0.12	2,630 (77.9)	707 (21.0)	38 (1.1)	0.21	Unclear
rs266729	*ADIPOQ*	3q27	C	G	0.26	1,860 (54.9)	1,300 (38.3)	231 (6.8)	0.85	Major
rs1648707	*ADIPOQ*	3q27	A	C	0.33	1,526 (45.0)	1,500 (44.2)	365 (10.8)	0.90	Minor
rs182052	*ADIPOQ*	3q27	G	A	0.33	1,519 (44.8)	1,501 (44.3)	371 (10.9)	0.99	Unclear
rs1342387	*ADIPOR1*	1q32.1	G	A	0.45	986 (29.1)	1,744 (51.5)	660 (19.5)	0.02	Major
rs7539542	*ADIPOR1*	1q32.1	C	G	0.30	1,637 (48.4)	1,440 (42.6)	305 (9.0)	0.65	Unclear
rs12733285	*ADIPOR1*	1q32.1	C	T	0.29	1,707 (50.4)	1,418 (41.9)	260 (7.7)	0.14	Major
rs1044471	*ADIPOR2*	12p13.31	C	T	0.46	981 (29.0)	1,681 (49.7)	723 (21.4)	0.95	Unclear
rs767870	*ADIPOR2*	12p13.31	T	C	0.18	2,280 (67.3)	1,013 (29.9)	97 (2.9)	0.22	Unclear

Abbreviations: ADIPOQ, adiponectin; ADIPOR1 and -2, adiponectin receptor 1 and 2; GHRHR, growth hormone releasing hormone receptor; HWE, Hardy-Weinberg equilibrium; IGF, insulin-like growth factor; IGF1, insulin-like growth factor 1; IGFBP3, insulin-like growth factor binding protein 3; IRS1 and -2, insulin receptor substrate 1 and 2; MAF, minor allele frequency; N, number of; PPARG, peroxisome proliferator-activated receptor gamma

^a^Frequencies are presented for men and women together because none of these germline genetic variants were located on sex chromosomes.

^b^A reference list on the basis of which the unfavorable allele was defined is included in the Supplementary Table 1.

**Table 2:** Baseline characteristics of male and female subcohort members in the Netherlands Cohort Study (1986–2002), according to tertiles of the genetic sum score of unfavorable alleles in genes related to the IGF pathway.

Baselinecharacteristics	Male subcohort						Female subcohort					
	Genetic sum score^a^,^b^						Genetic sum score^a^,^b^					
	Tertile 1		Tertile 2		Tertile 3		Tertile 1		Tertile 2		Tertile 3	
	N (%)	Mean(SD)	N (%)	Mean(SD)	N (%)	Mean(SD)	N (%)	Mean(SD)	N (%)	Mean(SD)	N (%)	Mean(SD)
Age, years		61.3 (4.1)		61.3 (4.3)		61.5 (4.1)		61.4 (4.2)		61.5 (4.3)		61.2 (4.3)
Family history of CRC (yes)	28 (4.8)		37 (6.1)		22 (6.0)		27 (4.8)		40 (7.3)		23 (5.8)	
Diabetes after age 30 (yes)	37 (3.7)		21 (3.5)		8 (2.2)		15 (2.7)		21 (3.8)		11 (2.8)	
Adult BMI, kg/m^2^		24.9 (2.6)		24.9 (2.7)		25.0 (2.5)		25.4 (3.9)		25.1 (3.5)		25.0 (3.2)
Adult trouser/skirt size (≥median)	318 (59.3)		351 (63.9)		208 (62.5)		316 (56.9)		308 (56.7)		230 (59.4)	
Height, cm		176.2 (6.5)		176.7 (6.4)		176.8 (7.2)		164.7 (6.4)		165.5 (6.1)		165.1 (5.8)
BMI at age 20, kg/m^2^		21.7 (2.3)		21.5 (2.4)		21.9 (2.5)		21.4 (2.7)		21.4 (2.8)		21.5 (2.9)
Occupational physical activity^c^												
<8 kJ/min	319 (60.7)		309 (58.4)		194 (59.3)							
8-12	134 (25.5)		144 (27.2)		97 (29.7)							
>12	73 (13.9)		76 (14.4)		36 (11.0)							
Non-occupational physical activity												
≤30 min/day	108 (18.5)		102 (17.2)		62 (17.0)		135 (24.4)		139 (25.5)		85 (21.6)	
>30-60	167 (28.6)		205 (34.5)		122 (33.4)		174 (31.5)		162 (29.7)		147 (37.3)	
>60-90	110 (18.8)		107 (18.0)		67 (18.4)		128 (23.2)		118 (21.6)		91 (23.1)	
>90	199 (34.1)		180 (30.3)		114 (31.2)		116 (21.0)		127 (23.3)		71 (18.0)	
Smoking status												
Never	84 (14.3)		75 (12.4)		42 (11.4)		326 (57.9)		322 (58.3)		234 (59.4)	
Ex	311 (52.9)		318 (52.7)		204 (55.3)		125 (22.2)		110 (19.9)		83 (21.1)	
Current	193 (32.8)		210 (34.8)		123 (33.3)		112 (19.9)		120 (21.7)		77 (19.5)	
Alcohol intake												
0 g/day	75 (12.9)		82 (13.9)		58 (15.7)		193 (35.9)		170 (31.9)		101 (27.0)	
0.1-29	412 (70.8)		422 (71.3)		262 (71.0)		330 (61.5)		350 (65.7)		252 (67.4)	
≥30	95 (16.3)		88 (14.9)		49 (13.3)		14 (2.6)		13 (2.4)		21 (5.6)	
Meat intake, g/day		105.9 (44.1)		106.2 (43.5)		102.0 (39.8)		91.9 (41.7)		90.5 (38.4)		98.1 (37.8)
Processed meat intake, g/day		16.8 (16.9)		17.3 (18.3)		16.1 (16.5)		10.3 (12.5)		11.3 (11.9)		11.3 (12.6)
Total energy intake, kcal/day		2,161 (493)		2,167 (507)		2,136 (464)		1,660 (399)		1,670 (370)		1,720 (389)

Abbreviations: BMI, body mass index; CRC, colorectal cancer; IGF, insulin-like growth factor; N, number of; SD, standard deviation

^a^The range in tertiles of the literature-based genetic sum score was 6–14, 15–18, and 19–29 unfavorable alleles. The theoretical maximum was 36.

^b^Chi-square and ANOVA tests showed no differences in most baseline characteristics between tertiles of the genetic sum score in men and women (P > 0.05), except for alcohol, meat, and total energy intake in women (P = 0.005, 0.01, and 0.048, respectively).

^c^Women in the Netherlands Cohort Study were mostly homemakers; few held jobs or only for a brief number of years in the distant past.

**Table 3:** Age-adjusted hazard ratios (HRs) and 95% confidence intervals (CIs) for colorectal cancer endpoints in relation to the genetic sum score of unfavorable alleles in genes related to the insulin-like growth factor pathway in men and women in the Netherlands Cohort Study (1986–2002).

	PY	Colorectal cancer			Colon cancer			Proximal colon cancer			Distal colon cancer			Rectal cancer		
		Ncases	HR	(95% CI)	Ncases	HR	(95% CI)	Ncases	HR	(95% CI)	Ncases	HR	(95% CI)	Ncases	HR	(95% CI)
**Men**																
Genetic sum score^a^																
Tertile 1	8,026	417	1	reference	272	1	reference	124	1	reference	139	1	reference	112	1	reference
Tertile 2	8,214	514	1.21	(1.01, 1.44)	319	1.15	(0.94, 1.40)	138	1.09	(0.83, 1.43)	167	1.18	(0.91, 1.52)	141	1.23	(0.94, 1.62)
Tertile 3	5,119	367	1.36	(1.11, 1.65)	246	1.39	(1.11, 1.74)	108	1.34	(1.00, 1.79)	133	1.48	(1.12, 1.94)	85	1.17	(0.86, 1.60)
*P*-trend			0.002			0.004			0.06			0.006			0.25	
Per unfavorable allele	19,968	1,201	1.03	(1.01, 1.05)	775	1.04	(1.02, 1.06)	342	1.04	(1.01, 1.08)	406	1.04	(1.01, 1.07)	312	1.01	(0.97,1.04)
*IGF1* CA repeat^b^																
19/19	5,457	402	1	reference	265	1	reference	116	1	reference	138	1	reference	103	1	reference
19/non-19	6,301	430	1.02	(0.81, 1.10)	289	0.92	(0.74, 1.15)	128	0.93	(0.70, 1.24)	149	0.92	(0.70, 1.20)	105	0.87	(0.64, 1.18)
Non-19/non-19	3,522	272	1.05	(1.03, 1.07)	168	0.96	(0.74, 1.24)	78	1.01	(0.72, 1.40)	88	0.97	(0.71, 1.32)	70	1.03	(0.73, 1.46)
*P*-trend			0.98			0.67			0.97			0.77			0.97	
Sum of *IGF1* CA repeats^c^																
≤36 repeats	2,051	160	1.12	(0.86, 1.47)	96	1.03	(0.76, 1.40)	42	1.04	(0.69, 1.56)	54	1.11	(0.77, 1.60)	48	1.36	(0.92, 2.01)
37	1,609	105	0.95	(0.70, 1.29)	69	0.96	(0.68, 1.35)	36	1.17	(0.76, 1.80)	31	0.81	(0.52, 1.27)	27	0.98	(0.61, 1.58)
38	6,330	434	1	reference	283	1	reference	123	1	reference	149	1	reference	108	1	reference
39	3,871	221	0.81	(0.65, 1.02)	150	0.85	(0.66, 1.09)	59	0.76	(0.54, 1.08)	85	0.91	(0.67, 1.24)	50	0.74	(0.51, 1.08)
≥40	2,459	184	1.06	(0.83, 1.37)	124	1.10	(0.83, 1.46)	62	1.26	(0.88, 1.81)	56	0.95	(0.66, 1.35)	45	1.05	(0.71, 1.56)
*P*-trend			0.36			0.89			0.96			0.58			0.11	
**Women**																
Genetic sum score^a^																
Tertile 1	8,434	279	1	reference	213	1	reference	124	1	reference	82	1	reference	47	1	reference
Tertile 2	8,235	343	1.25	(1.02, 1.52)	256	1.22	(0.98, 1.52)	150	1.22	(0.93, 1.60)	100	1.24	(0.91, 1.70)	59	1.28	(0.85, 1.91)
Tertile 3	5,927	234	1.21	(0.97, 1.50)	166	1.12	(0.88, 1.43)	90	1.05	(0.77, 1.42)	70	1.22	(0.87, 1.73)	49	1.50	(0.98, 2.28)
*P*-trend			0.07			0.28			0.63			0.22			0.06	
Per unfavorable allele	20,760	762	1.01	(0.99, 1.04)	564	1.00	(0.98, 1.03)	326	1.01	(0.98, 1.04)	220	0.99	(0.96, 1.03)	138	1.04	(0.99, 1.09)
*IGF1* CA repeat^b^																
19/19	4,323	264	1	reference	209	1	reference	117	1	reference	89	1	reference	35	1	reference
19/non-19	5,535	290	0.84	(0.67, 1.06)	217	0.79	(0.62, 1.02)	133	0.86	(0.64, 1.16)	77	0.67	(0.47, 0.94)	52	1.14	(0.72, 1.81)
Non-19/non-19	5,313	175	0.54	(0.42, 0.70)	127	0.50	(0.38, 0.65)	68	0.48	(0.34, 0.67)	57	0.52	(0.36, 0.75)	36	0.84	(0.52, 1.38)
*P*-trend			<0.001			<0.001			<0.001			0.001			0.46	
Sum of *IGF1* CA repeats^c^																
≤36 repeats	4,061	95	0.44	(0.33, 0.58)	71	0.41	(0.30, 0.56)	40	0.41	(0.28, 0.61)	30	0.41	(0.27, 0.64)	17	0.55	(0.30, 0.98)
37	2,228	81	0.67	(0.49, 0.92)	58	0.61	(0.43, 0.87)	34	0.64	(0.42, 0.98)	21	0.52	(0.31, 0.87)	18	1.05	(0.58, 1.88)
38	5,477	294	1	reference	231	1	reference	130	1	reference	98	1	reference	42	1	reference
39	2,770	164	1.09	(0.84, 1.42)	126	1.07	(0.80, 1.42)	81	1.22	(0.87, 1.70)	43	0.86	(0.58, 1.29)	27	1.26	(0.76, 2.11)
≥40	1,780	95	0.99	(0.72, 1.35)	67	0.89	(0.63, 1.25)	33	0.77	(0.50, 1.20)	31	0.97	(0.61, 1.52)	19	1.39	(0.77, 2.48)
*P*-trend			<0.001			<0.001			<0.001			<0.001			0.004	

Abbreviations: CI, confidence interval; HR, hazard ratio; IGF1, insulin-like growth factor 1; N, number of; PY, person-years at risk.

^a^The range in tertiles of the literature-based genetic sum score was 6–14, 15–18, and 19–29 unfavorable alleles. The theoretical maximum was 36. The distribution of subcohort members across tertiles was 588 (37.7%), 603 (38.7%), and 369 (23.7%), respectively, in men and 563 (37.3%), 552 (36.6%), and 394 (26.1%), respectively, in women.

^b^The distribution of subcohort members across categories of the *IGF1* CA-repeat variable was 414 (36.8%), 452 (40.2%), and 258 (23.0%), respectively, in men and 293 (29.0%), 366 (36.2%), and 351 (34.8%), respectively, in women.

^c^The distribution of subcohort members across categories of the *IGF1* CA-repeat variable was 143 (12.7%), 107 (9.5%), 449 (40.0%), 256 (22.8%), and 169 (15.0%), respectively, in men and 253 (25.1%), 136 (13.5%), 347 (34.4%), 170 (16.8%), and 104 (10.3%), respectively, in women.

**Table 4:** Age-adjusted Hazard Ratios (HR) and 95% Confidence Intervals (CI) for Colorectal Cancer in Relation to SNPs in Genes in the Insulin-like Growth Factor Pathway in Men and Women in the Netherlands Cohort Study (1986–2002).

	Colorectal cancer							
	Men				Women			
	PY	N cases	HR	(95% CI)	PY	N cases	HR	(95% CI)
***IGF1***								
rs1520220								
CC	15,286	895	1	reference	15,700	603	1	reference
CG	6,178	414	1.15	(0.97, 1.35)	7,207	277	1.00	(0.83, 1.19)
GG	704	54	1.37	(0.92, 2.05)	765	31	1.03	(0.65, 1.63)
*P*-trend			0.04				0.98	
rs5742678								
CC	12,877	745	1	reference	13,025	497	1	reference
GC	7,912	519	1.14	(0.98, 1.34)	9,086	355	1.01	(0.85, 1.20)
GG	1,379	98	1.25	(0.92, 1.69)	1,561	59	0.98	(0.69, 1.37)
*P*-trend			0.05				0.97	
rs5742694								
TT	13,469	778	1	reference	13,695	518	1	reference
GT	7,462	487	1.13	(0.97, 1.33)	8,568	341	1.03	(0.86, 1.22)
GG	1,237	96	1.38	(1.01, 1.88)	1,410	51	0.96	(0.67, 1.37)
*P*-trend			0.02				0.95	
rs10735380								
AA	12,365	724	1	reference	12,983	469	1	reference
AG	8,346	533	1.10	(0.94, 1.28)	9,052	378	1.14	(0.96, 1.35)
GG	1,456	105	1.25	(0.93, 1.67)	1,638	64	1.06	(0.76, 1.47)
*P*-trend			0.09				0.26	
***IGFBP3***								
rs2854744								
AA	4,654	279	1	reference	4,778	193	1	reference
CA	11,173	690	1.04	(0.85, 1.26)	11,750	446	0.93	(0.75, 1.15)
CC	6,341	393	1.03	(0.83, 1.28)	7,128	270	0.96	(0.76, 1.22)
*P*-trend			0.80				0.82	
rs2132572								
GG	13,360	768	1	reference	13,901	518	1	reference
GA	7,661	510	1.12	(0.96, 1.31)	8,476	343	1.07	(0.90, 1.27)
AA	1,147	85	1.29	(0.93, 1.79)	1,295	50	1.06	(0.73, 1.54)
*P*-trend			0.06				0.49	
rs35440925								
CC	13,704	882	1	reference	14,615	579	1	reference
CG	7,479	422	0.89	(0.76, 1.05)	8,113	294	0.92	(0.77, 1.10)
GG	985	59	0.97	(0.67, 1.39)	944	37	1.02	(0.67, 1.57)
*P*-trend			0.27				0.54	
***IRS1***								
rs1801278								
GG	19,004	1,155	1	reference	20,576	789	1	reference
GA	3,017	203	1.11	(0.90, 1.37)	3,047	119	1.03	(0.80, 1.31)
AA	147	5	0.60	(0.20, 1.77)	49	3	2.33	(0.47, 11.65)
*P*-trend			0.58				0.66	
***IRS2***								
rs1805097								
AA	2,709	151	1	reference	3,018	111	1	reference
GA	10,044	604	1.07	(0.84, 1.36)	10,621	405	1.01	(0.77, 1.32)
GG	9,274	603	1.15	(0.90, 1.47)	9,985	392	1.07	(0.82, 1.40)
*P*-trend			0.20				0.51	
rs2289046								
GG	2,511	134	1	reference	2,838	109	1	reference
GA	10,059	607	1.13	(0.88, 1.46)	10,676	398	0.94	(0.72, 1.23)
AA	9,598	622	1.20	(0.93, 1.55)	10,158	404	1.03	(0.78, 1.34)
*P*-trend			0.15				0.57	
rs754204								
CC	5,876	336	1	reference	6,496	248	1	reference
TC	11,128	694	1.08	(0.91, 1.30)	11,468	438	0.98	(0.81, 1.20)
TT	4,754	326	1.17	(0.95, 1.45)	5,291	215	1.04	(0.82, 1.31)
*P*-trend			0.14				0.78	
rs4773082								
TT	6,560	357	1	reference	6,954	249	1	reference
CT	10,190	623	1.11	(0.93, 1.33)	10,299	382	1.00	(0.82, 1.23)
CC	3,813	243	1.12	(0.89, 1.41)	4,082	152	1.03	(0.80, 1.33)
*P*-trend			0.27				0.81	
***GHRHR***								
rs4988496								
GG	19,743	1,204	1	reference	20,791	813	1	reference
AG	2,346	153	1.05	(0.83, 1.33)	2,783	93	0.86	(0.66, 1.12)
AA	63	4	1.02	(0.25, 4.10)	98	5	1.32	(0.40, 4.38)
*P*-trend			0.72				0.39	
***PPARG***								
rs1801282								
GG	187	12	1	reference	389	12	1	reference
GC	4,814	280	0.86	(0.38, 1.95)	4,916	194	1.26	(0.61, 2.58)
CC	17,151	1,071	0.92	(0.41, 2.06)	18,363	705	1.21	(0.60, 2.44)
*P*-trend			0.58				0.93	
***ADIPOQ***								
rs1501299								
AA	1,620	103	1	reference	1,710	51	1	reference
CA	8,834	108	0.92	(0.68, 1.23)	8,779	366	1.39	(0.97, 1.98)
CC	11,715	751	1.03	(0.77, 1.37)	13,184	493	1.26	(0.89, 1.79)
*P*-trend			0.34				0.88	
rs2241766								
TT	17,305	1,103	1	reference	18,368	729	1	reference
GT	4,562	244	0.83	(0.69, 1.00)	4,849	164	0.85	(0.68, 1.05)
GG	170	10	0.91	(0.38, 2.16)	358	16	1.10	(0.57, 2.12)
*P*-trend			0.06				0.25	
rs266729								
GG	1,329	93	1	reference	1,909	60	1	reference
GC	8,742	545	0.90	(0.66, 1.23)	8,479	334	1.32	(0.94, 1.86)
CC	12,098	724	0.84	(0.62, 1.15)	13,285	517	1.29	(0.93, 1.80)
*P*-trend			0.22				0.36	
rs1648707								
AA	9,974	597	1	reference	10,948	426	1	reference
CA	10,041	616	1.03	(0.88, 1.21)	9,970	393	1.00	(0.84, 1.19)
CC	2,153	150	1.19	(0.92, 1.53)	2,754	92	0.81	(0.62, 1.08)
*P*-trend			0.25				0.27	
rs182052								
GG	9,900	592	1	reference	10,891	424	1	reference
GA	10,085	617	1.03	(0.88, 1.21)	9,966	393	1.00	(0.84, 1.19)
AA	2,183	154	1.20	(0.93, 1.54)	2,816	94	0.82	(0.62, 1.08)
*P*-trend			0.23				0.29	
***ADIPOR1***								
rs1342387								
AA	4,266	234	1	reference	4,555	195	1	reference
GA	11,418	725	1.16	(0.95, 1.42)	12,350	447	0.86	(0.69, 1.06)
GG	6,484	403	1.12	(0.90, 1.40)	6,751	267	0.94	(0.74, 1.20)
*P*-trend			0.42				0.77	
rs7539542								
CC	10,697	671	1	reference	11,152	469	1	reference
CG	9,496	566	0.95	(0.82, 1.11)	10,282	361	0.84	(0.71, 1.00)
GG	1,957	125	0.99	(0.76, 1.30)	2,130	81	0.95	(0.70, 1.29)
*P*-trend			0.72				0.19	
rs12733285								
CC	1,884	94	1	reference	1,610	80	1	reference
CT	9,301	573	1.27	(0.95, 1.70)	9,981	370	0.76	(0.56, 1.05)
TT	10,971	696	1.29	(0.97, 1.72)	12,008	459	0.80	(0.58, 1.09)
*P*-trend			0.19				0.51	
***ADIPOR2***								
rs1044471								
CC	6,286	409	1	reference	6,861	240	1	reference
CT	11,233	660	0.90	(0.76, 1.07)	11,637	473	1.12	(0.92, 1.36)
TT	4,614	293	0.98	(0.79, 1.21)	5,141	197	1.08	(0.85, 1.37)
*P*-trend			0.74				0.47	
rs767870								
TT	14,593	923	1	reference	16,325	609	1	reference
TC	6,917	403	0.91	(0.77, 1.07)	6,664	278	1.13	(0.94, 1.35)
CC	659	37	0.87	(0.56, 1.36)	668	23	0.93	(0.56, 1.57)
*P*-trend			0.22				0.36	

Abbreviations: ADIPOQ, adiponectin; ADIPOR1 and -2, adiponectin receptor 1 and 2; CI, confidence interval; GHRHR, growth hormone releasing hormone receptor; HR, hazard ratio; IGF1, insulin-like growth factor 1; IGFBP3, insulin-like growth factor binding protein 3; IRS1 and -2, insulin receptor substrate 1 and 2; N, number of; PPARG, peroxisome proliferator-activated receptor gamma; PY, person-years at risk.
