# Supplementary material for: Genetic Variants in the Insulin-like Growth Factor Pathway and Colorectal Cancer Risk in the Netherlands Cohort Study
Source: Sci Rep. 2015 Sep 18;5:14126. doi: 10.1038/srep14126 (PMC4585376; doi:10.1038/srep14126)
Supplement: Supplementary Information [file srep14126-s1.doc]

**Genetic Variants in the Insulin-like Growth Factor Pathway and Colorectal Cancer Risk in the Netherlands Cohort Study**

Simons Colinda CJM^1^, Schouten Leo J^1^, Godschalk Roger WL^2^, van Engeland Manon^3^, van den Brandt Piet A^1^, van Schooten Frederik J.^2^, Weijenberg Matty P^1^

^1^ Department of Epidemiology, GROW – School for Oncology and Developmental

Biology, Maastricht University, Maastricht, the Netherlands

^2^ Department of Toxicology, NUTRIM – School of Nutrition and Translational Research in Metabolism, Maastricht University, Maastricht, the Netherlands

^3^ Department of Pathology, GROW – School for Oncology and Developmental Biology, Maastricht University Medical Centre, Maastricht, the Netherlands

| **Supplementary Table 1.** References on the basis of which single nucleotide polymorphisms in genes related to the insulin-like growth factor pathway were scored and selected for genotyping, and on the basis of which the unfavorable allele was discerned | | | | | | | | |
| --- | --- | --- | --- | --- | --- | --- | --- | --- |
| SNP | Gene | Type of  variant | Score on points  i/ii/iii/iv/v/vi/vii^a^ | Total  selection  score | Major  allele | Minor  allele | Unfavorable  allele as based  on literature | References^b^ |
| rs1520220 | *IGF1* | Intron | 0/2/0/1/0/0/0 | 3 | C | G | Minor | 1–4 |
| rs5742678 | *IGF1* | Intron | 0/1/0/1/0/0/0 | 2 | C | G | Minor | 1 |
| rs5742694 | *IGF1* | Intron | 0/1/0/1/0/0/0 | 2 | T | G | Minor | 1 |
| rs10735380 | *IGF1* | Intron | 0/2/0/0/0/0/0 | 2 | A | G | Minor | 2,3 |
| rs2854744 | *IGFBP3* | Intron | 2/2/0/2/0/0/0 | 6 | C | A | Major | 1–24 |
| rs2132572 | *IGFBP3* | Upstream | 1/2/0/1/0/0/0 | 4 | G | A | Minor | 1,13,16,22,24 |
| rs35440925 | *IGFBP3* | Intron | 0/2/0/0/0/0/0 | 2 | C | G | Unclear | Proxy for rs2471551^1,13,24,25^ |
| rs1801278 | *IRS1* | Missense | 2/2/2/1/0/0/0 | 7 | G | A | Minor | 6,8,10,14,17,26–35 |
| rs1805097 | *IRS2* | Missense | 2/2/2/2/0/0/0 | 8 | G | A | Major | 6,8,10,14,17,26,27,30,32,35–43 |
| rs2289046 | *IRS2* | 3’ Prime UTR | 0/1/0/1/0/0/0 | 2 | A | G | Major | 44 |
| rs754204 | *IRS2* | Intron | 0/1/0/1/0/0/0 | 2 | C | T | Minor | 44 |
| rs4773082 | *IRS2* | Downstream | 0/1/0/1/0/0/0 | 2 | T | C | Minor | 44 |
| rs4988496 | *GHRHR* | Missense | 0/0/0/0/0/0/0 | 0 | G | A | Minor | 45 |
| rs1801282 | *PPARG* | Missense | 2/2/2/2/0/2/0 | 10 | C | G | Major | 29,35,46–76 |
| rs1501299 | *ADIPOQ* | Intron | 2/2/0/2/0/0/0 | 6 | C | A | Major | 66,75,77–89 |
| rs2241766 | *ADIPOQ* | Synonymous | 0/2/1/2/0/0/0 | 5 | T | G | Unclear | 76–78,80–90 |
| rs266729 | *ADIPOQ* | Upstream | 2/2/1/0/0/0/0 | 5 | C | G | Major | 77,80–84,87,91–96 |
| rs1648707 | *ADIPOQ* | Intergenic | 1/0/0/0/0/0/2 | 3 | A | C | Minor | 91,97 |
| rs182052 | *ADIPOQ* | Intron | 0/2/0/1/0/0/0 | 3 | G | A | Unclear | 86,96 |
| rs1342387 | *ADIPOR1* | Intron | 2/2/2/0/0/0/0 | 6 | G | A | Major | 77,80–84,87,95,98–106 |
| rs7539542 | *ADIPOR1* | 3’ Prime UTR | 0/2/0/1/0/0/0 | 3 | C | G | Unclear | 77,80–82,84,95,98,101–103,106–108 |
| rs12733285 | *ADIPOR1* | Intron | 1/0/1/0/0/0/0 | 2 | C | T | Major | 80–82,87,99,105,106 |
| rs1044471 | *ADIPOR2* | 3’ Prime UTR | 0/1/2/0/0/0/0 | 3 | C | T | Unclear | 77,82,83,95,98,99,102,103,105,106,108 |
| rs767870 | *ADIPOR2* | Intron | 0/0/2/0/0/0/0 | 2 | T | C | Unclear | 77,102,103 |
| Abbreviations: ADIPOQ, adiponectin; ADIPOR1 and -2, adiponectin receptor 1 and 2; GHRHR, growth hormone releasing hormone receptor; HWE, Hardy-Weinberg equilibrium; IGF1, insulin-like growth factor 1; IGFBP3, insulin-like growth factor binding protein 3; IRS1 and -2, insulin receptor substrate 1 and 2; MAF, minor allele frequency; PPARG, peroxisome proliferator-activated receptor gamma  ^a^ Scores on points i–iv indicate whether the SNP was found associated with i) CRC risk, ii) obesity, insulin resistance or blood levels of IGF pathway-related factors, iii) type 2 diabetes mellitus risk, or iv) the risk of cancers of the oesophagus, pancreas, gallbladder, breast (in postmenopausal women), endometrium, and kidney (which are considered to be obesity-related cancers) in one or more studies in the literature. Scores on points v–vii indicate whether the SNP was found associated with v) CRC, vi) type 2 diabetes, or vii) obesity, insulin resistance or blood levels of IGF pathway-related factors in one or more genome-wide association studies.  ^b^ A reference list is included at the end of the document. | | | | | | | | |

| **Supplementary Table 2.** Age-adjusted Hazard ratios (HR) and 95% Confidence Intervals (CI) for Colorectal Cancer Endpoints in Relation to the Number of Genes in the Insulin-like Growth Factor Pathway with Unfavorable Alleles in Men and Women in the Netherlands Cohort Study (1986–2002) | | | | | | | | | | | | | | | | |
| --- | --- | --- | --- | --- | --- | --- | --- | --- | --- | --- | --- | --- | --- | --- | --- | --- |
|  |  | Colorectal cancer | | | Colon cancer | | | Proximal colon cancer | | | Distal colon cancer | | | Rectal cancer | | |
|  | PY | N cases  cases | HR | (95% CI) | N cases  cases | HR | (95% CI) | N cases  cases | HR | (95% CI) | N cases  cases | HR | (95% CI) | N cases  cases | HR | (95% CI) |
| Number of genes  with unfavorable  alleles (range 0-8) |  |  |  |  |  |  |  |  |  |  |  |  |  |  |  |  |
| Men | 29,718 | 1,363 | 1.46 | (1.40, 1.51) | 879 | 1.47 | (1.41, 1.53) | 391 | 1.47 | (1.39, 1.55) | 459 | 1.46 | (1.39, 1.54) | 355 | 1.44 | (1.37, 1.52) |
| Women | 33,221 | 911 | 1.42 | (1.38, 1.48) | 677 | 1.41 | (1.36, 1.47) | 392 | 1.41 | (1.35, 1.47) | 265 | 1.42 | (1.34, 1.49) | 163 | 1.48 | (1.38, 1.59) |
| Abbreviations: CI, confidence interval; HR, hazard ratio; N, number of; PY, person-years at risk | | | | | | | | | | | | | | | | |

**REFERENCES**

1. Al-Zahrani, A. *et al.* IGF1 and IGFBP3 tagging polymorphisms are associated with circulating levels of IGF1, IGFBP3 and risk of breast cancer. *Hum. Mol. Genet.* **15,** 1–10 (2006).

2. Patel, A. V. *et al.* IGF-1, IGFBP-1, and IGFBP-3 polymorphisms predict circulating IGF levels but not breast cancer risk: findings from the Breast and Prostate Cancer Cohort Consortium (BPC3). *PloS One* **3,** e2578 (2008).

3. Gu, F. *et al.* Eighteen insulin-like growth factor pathway genes, circulating levels of IGF-I and its binding protein, and risk of prostate and breast cancer. *Cancer Epidemiol. Biomark. Prev.* **19,** 2877–2887 (2010).

4. McGrath, M., Lee, I.-M., Buring, J. & De Vivo, I. Common genetic variation within IGFI, IGFII, IGFBP-1, and IGFBP-3 and endometrial cancer risk. *Gynecol. Oncol.* **120,** 174–178 (2011).

5. Deal, C. *et al.* Novel promoter polymorphism in insulin-like growth factor-binding protein-3: correlation with serum levels and interaction with known regulators. *J. Clin. Endocrinol. Metab.* **86,** 1274–1280 (2001).

6. Slattery, M. L. *et al.* Associations among IRS1, IRS2, IGF1, and IGFBP3 genetic polymorphisms and colorectal cancer. *Cancer Epidemiol Biomark. Prev* **13,** 1206–14 (2004).

7. Le Marchand, L., Kolonel, L. N., Henderson, B. E. & Wilkens, L. R. Association of an exon 1 polymorphism in the IGFBP3 gene with circulating IGFBP-3 levels and colorectal cancer risk: the multiethnic cohort study. *Cancer Epidemiol. Biomark. Prev.* **14,** 1319–1321 (2005).

8. Slattery, M. L. *et al.* Energy balance, insulin-related genes and risk of colon and rectal cancer. *Int J Cancer* **115,** 148–54 (2005).

9. Slattery, M. L. *et al.* Genetic, anthropometric, and lifestyle factors associated with IGF-1 and IGFBP-3 levels in Hispanic and non-Hispanic white women. *Cancer Causes Control* **16,** 1147–1157 (2005).

10. Sweeney, C. *et al.* Insulin-like growth factor pathway polymorphisms associated with body size in Hispanic and non-Hispanic white women. *Cancer Epidemiol. Biomark. Prev.* **14,** 1802–1809 (2005).

11. Wagner, K. *et al.* Polymorphisms in the IGF-1 and IGFBP 3 promoter and the risk of breast cancer. *Breast Cancer Res. Treat.* **92,** 133–140 (2005).

12. Wong, H. L. *et al.* A new single nucleotide polymorphism in the insulin-like growth factor I regulatory region associates with colorectal cancer risk in singapore chinese. *Cancer Epidemiol Biomark. Prev* **14,** 144–51 (2005).

13. Canzian, F. *et al.* Polymorphisms of genes coding for insulin-like growth factor 1 and its major binding proteins, circulating levels of IGF-I and IGFBP-3 and breast cancer risk: results from the EPIC study. *Br. J. Cancer* **94,** 299–307 (2006).

14. Samowitz, W. S. *et al.* Polymorphisms in insulin-related genes predispose to specific KRAS2 and TP53 mutations in colon cancer. *Mutat Res* **595,** 117–24 (2006).

15. Cheng, I. *et al.* Genetic determinants of circulating insulin-like growth factor (IGF)-I, IGF binding protein (BP)-1, and IGFBP-3 levels in a multiethnic population. *J. Clin. Endocrinol. Metab.* **92,** 3660–3666 (2007).

16. Pechlivanis, S. *et al.* Polymorphisms in the insulin like growth factor 1 and IGF binding protein 3 genes and risk of colorectal cancer. *Cancer Detect Prev* **31,** 408–16 (2007).

17. Slattery, M. L. *et al.* Genetic variation in IGF1, IGFBP3, IRS1, IRS2 and risk of breast cancer in women living in Southwestern United States. *Breast Cancer Res. Treat.* **104,** 197–209 (2007).

18. Chen, W. *et al.* Phenotypes and genotypes of insulin-like growth factor 1, IGF-binding protein-3 and cancer risk: evidence from 96 studies. *Eur. J. Hum. Genet.* **17,** 1668–1675 (2009).

19. Mong, J. L. Y. *et al.* Associations of insulin-like growth factor binding protein-3 gene polymorphisms with IGF-I activity and lipid parameters in adolescents. *Int. J. Obes. 2005* **33,** 1446–1453 (2009).

20. Xiang, H. *et al.* Association between two functional polymorphisms of insulin-like growth factor binding protein 3 and colorectal cancer risk in a Chinese population. *J Toxicol Env. Health A* **72,** 706–11 (2009).

21. Feik, E. *et al.* Association of IGF1 and IGFBP3 polymorphisms with colorectal polyps and colorectal cancer risk. *Cancer Causes Control* **21,** 91–97 (2010).

22. Taverne, C. W. *et al.* Common genetic variation of insulin-like growth factor-binding protein 1 (IGFBP-1), IGFBP-3, and acid labile subunit in relation to serum IGF-I levels and mammographic density. *Breast Cancer Res. Treat.* **123,** 843–855 (2010).

23. Qiu, L.-X. *et al.* IGFBP3 A-202C polymorphism and breast cancer susceptibility: a meta-analysis involving 33,557 cases and 45,254 controls. *Breast Cancer Res. Treat.* **122,** 867–871 (2010).

24. Rosendahl, A. H., Hietala, M., Henningson, M., Olsson, H. & Jernström, H. IGFBP1 and IGFBP3 polymorphisms predict circulating IGFBP-3 levels among women from high-risk breast cancer families. *Breast Cancer Res. Treat.* **127,** 785–794 (2011).

25. Biong, M. *et al.* Genotypes and haplotypes in the insulin-like growth factors, their receptors and binding proteins in relation to plasma metabolic levels and mammographic density. *BMC Med. Genomics* **3,** 9 (2010).

26. Le Fur, S., Le Stunff, C. & Bougnères, P. Increased insulin resistance in obese children who have both 972 IRS-1 and 1057 IRS-2 polymorphisms. *Diabetes* **51 Suppl 3,** S304–307 (2002).

27. ’t Hart, L. M. *et al.* Variations in insulin secretion in carriers of gene variants in IRS-1 and -2. *Diabetes* **51,** 884–887 (2002).

28. Jellema, A., Zeegers, M. P. A., Feskens, E. J. M., Dagnelie, P. C. & Mensink, R. P. Gly972Arg variant in the insulin receptor substrate-1 gene and association with Type 2 diabetes: a meta-analysis of 27 studies. *Diabetologia* **46,** 990–995 (2003).

29. Florez, J. C. *et al.* Association testing in 9,000 people fails to confirm the association of the insulin receptor substrate-1 G972R polymorphism with type 2 diabetes. *Diabetes* **53,** 3313–3318 (2004).

30. Laukkanen, O. *et al.* Common polymorphisms in the genes regulating the early insulin signalling pathway: effects on weight change and the conversion from impaired glucose tolerance to Type 2 diabetes. The Finnish Diabetes Prevention Study. *Diabetologia* **47,** 871–877 (2004).

31. Zeggini, E. *et al.* Association studies of insulin receptor substrate 1 gene (IRS1) variants in type 2 diabetes samples enriched for family history and early age of onset. *Diabetes* **53,** 3319–3322 (2004).

32. Pechlivanis, S. *et al.* Insulin pathway related genes and risk of colorectal cancer: INSR promoter polymorphism shows a protective effect. *Endocr. Relat. Cancer* **14,** 733–740 (2007).

33. Morini, E. *et al.* IRS1 G972R polymorphism and type 2 diabetes: a paradigm for the difficult ascertainment of the contribution to disease susceptibility of ‘low-frequency-low-risk’ variants. *Diabetologia* **52,** 1852–1857 (2009).

34. Neuhausen, S. L. *et al.* Genetic variation in insulin-like growth factor signaling genes and breast cancer risk among BRCA1 and BRCA2 carriers. *Breast Cancer Res.* **11,** R76 (2009).

35. Slattery, M. L. *et al.* Colon tumor mutations and epigenetic changes associated with genetic polymorphism: insight into disease pathways. *Mutat Res* **660,** 12–21 (2009).

36. Mammarella, S. *et al.* Interaction between the G1057D variant of IRS-2 and overweight in the pathogenesis of type 2 diabetes. *Hum. Mol. Genet.* **9,** 2517–2521 (2000).

37. D’Alfonso, R. *et al.* Polymorphisms of the insulin receptor substrate-2 in patients with type 2 diabetes. *J. Clin. Endocrinol. Metab.* **88,** 317–322 (2003).

38. Stefan, N. *et al.* Metabolic effects of the Gly1057Asp polymorphism in IRS-2 and interactions with obesity. *Diabetes* **52,** 1544–1550 (2003).

39. Okazawa, K. *et al.* The haplotypes of the IRS-2 gene affect insulin sensitivity in Japanese patients with type 2 diabetes. *Diabetes Res. Clin. Pract.* **68,** 39–48 (2005).

40. Kong, L. *et al.* Study on the relationship between G1057D variants of IRS2 gene and obese T2DM in Chinese Han subjects. *Chin. J. Med. Genet.* **22,** 387–390 (2005).

41. Slattery, M. L., Wolff, R. K., Herrick, J., Caan, B. J. & Potter, J. D. Leptin and leptin receptor genotypes and colon cancer: gene-gene and gene-lifestyle interactions. *Int J Cancer* **122,** 1611–7 (2008).

42. Ouederni, T. B. *et al.* The G1057D polymorphism of IRS-2 gene is not associated with type 2 diabetes and obese patients among ethnic groups in Tunisian population. *Clin. Biochem.* **42,** 1169–1173 (2009).

43. Cayan, F. *et al.* Insulin receptor substrate-2 gene polymorphism: is it associated with endometrial cancer? *Gynecol. Endocrinol. Off. J. Int. Soc. Gynecol. Endocrinol.* **26,** 378–382 (2010).

44. Feigelson, H. S. *et al.* Genetic variation in candidate obesity genes ADRB2, ADRB3, GHRL, HSD11B1, IRS1, IRS2, and SHC1 and risk for breast cancer in the Cancer Prevention Study II. *Breast Cancer Res.* **10,** R57 (2008).

45. Canzian, F. *et al.* Comprehensive analysis of common genetic variation in 61 genes related to steroid hormone and insulin-like growth factor-I metabolism and breast cancer risk in the NCI breast and prostate cancer cohort consortium. *Hum. Mol. Genet.* **19,** 3873–3884 (2010).

46. Smith, W. M. *et al.* Opposite association of two PPARG variants with cancer: overrepresentation of H449H in endometrial carcinoma cases and underrepresentation of P12A in renal cell carcinoma cases. *Hum. Genet.* **109,** 146–151 (2001).

47. Landi, S. *et al.* Association of common polymorphisms in inflammatory genes interleukin (IL)6, IL8, tumor necrosis factor alpha, NFKB1, and peroxisome proliferator-activated receptor gamma with colorectal cancer. *Cancer Res.* **63,** 3560–3566 (2003).

48. Paynter, R. A., Hankinson, S. E., Colditz, G. A., Hunter, D. J. & De Vivo, I. No evidence of a role for PPARgamma Pro12Ala polymorphism in endometrial cancer susceptibility. *Pharmacogenetics* **14,** 851–856 (2004).

49. Jiang, J. *et al.* Influence of the C161T but not Pro12Ala polymorphism in the peroxisome proliferator-activated receptor-gamma on colorectal cancer in an Indian population. *Cancer Sci* **96,** 507–12 (2005).

50. McGreavey, L. E. *et al.* No evidence that polymorphisms in CYP2C8, CYP2C9, UGT1A6, PPARdelta and PPARgamma act as modifiers of the protective effect of regular NSAID use on the risk of colorectal carcinoma. *Pharmacogenet. Genomics* **15,** 713–721 (2005).

51. Paracchini, V., Pedotti, P. & Taioli, E. Genetics of leptin and obesity: a HuGE review. *Am. J. Epidemiol.* **162,** 101–114 (2005).

52. Slattery, M. L. *et al.* PPARgamma, energy balance, and associations with colon and rectal cancer. *Nutr Cancer* **51,** 155–61 (2005).

53. Koh, W. P., Yuan, J. M., Van Den Berg, D., Ingles, S. A. & Yu, M. C. Peroxisome proliferator-activated receptor (PPAR) gamma gene polymorphisms and colorectal cancer risk among Chinese in Singapore. *Carcinogenesis* **27,** 1797–802 (2006).

54. Kuriki, K. *et al.* Meat, milk, saturated fatty acids, the Pro12Ala and C161T polymorphisms of the PPARgamma gene and colorectal cancer risk in Japanese. *Cancer Sci.* **97,** 1226–1235 (2006).

55. Tönjes, A., Scholz, M., Loeffler, M. & Stumvoll, M. Association of Pro12Ala polymorphism in peroxisome proliferator-activated receptor gamma with Pre-diabetic phenotypes: meta-analysis of 57 studies on nondiabetic individuals. *Diabetes Care* **29,** 2489–2497 (2006).

56. Scott, L. J. *et al.* A genome-wide association study of type 2 diabetes in Finns detects multiple susceptibility variants. *Science* **316,** 1341–1345 (2007).

57. Slattery, M. L. *et al.* PPARgamma and colon and rectal cancer: associations with specific tumor mutations, aspirin, ibuprofen and insulin-related genes (United States). *Cancer Causes Control* **17,** 239–49 (2006).

58. Theodoropoulos, G. *et al.* Relation between common polymorphisms in genes related to inflammatory response and colorectal cancer. *World J Gastroenterol* **12,** 5037–43 (2006).

59. Ludovico, O. *et al.* Heterogeneous effect of peroxisome proliferator-activated receptor gamma2 Ala12 variant on type 2 diabetes risk. *Obes.* **15,** 1076–1081 (2007).

60. Vogel, U. *et al.* Prospective study of interaction between alcohol, NSAID use and polymorphisms in genes involved in the inflammatory response in relation to risk of colorectal cancer. *Mutat. Res.* **624,** 88–100 (2007).

61. Vogel, U. *et al.* Peroxisome proliferator-activated [corrected] receptor-gamma2 [corrected] Pro12Ala, interaction with alcohol intake and NSAID use, in relation to risk of breast cancer in a prospective study of Danes. *Carcinogenesis* **28,** 427–434 (2007).

62. Wang, Y. *et al.* Nested case-control study of energy regulation candidate gene single nucleotide polymorphisms and breast cancer. *Anticancer Res.* **27,** 589–593 (2007).

63. Kury, S. *et al.* Low-penetrance alleles predisposing to sporadic colorectal cancers: a French case-controlled genetic association study. *BMC Cancer* **8,** 326 (2008).

64. Fesinmeyer, M. D. *et al.* Association between the peroxisome proliferator-activated receptor gamma Pro12Ala variant and haplotype and pancreatic cancer in a high-risk cohort of smokers: a pilot study. *Pancreas* **38,** 631–637 (2009).

65. Salanti, G. *et al.* Underlying genetic models of inheritance in established type 2 diabetes associations. *Am. J. Epidemiol.* **170,** 537–545 (2009).

66. Tsilidis, K. K. *et al.* Association of common polymorphisms in IL10, and in other genes related to inflammatory response and obesity with colorectal cancer. *Cancer Causes Control* **20,** 1739–51 (2009).

67. Gouda, H. N. *et al.* The association between the peroxisome proliferator-activated receptor-gamma2 (PPARG2) Pro12Ala gene variant and type 2 diabetes mellitus: a HuGE review and meta-analysis. *Am. J. Epidemiol.* **171,** 645–655 (2010).

68. Huguenin, G. V. B. & Rosa, G. The Ala allele in the PPAR-gamma2 gene is associated with reduced risk of type 2 diabetes mellitus in Caucasians and improved insulin sensitivity in overweight subjects. *Br. J. Nutr.* **104,** 488–497 (2010).

69. Laakso, S., Utriainen, P., Laakso, M., Voutilainen, R. & Jääskeläinen, J. Polymorphism Pro12Ala of PPARG in prepubertal children with premature adrenarche and its association with growth in healthy children. *Horm. Res. Pædiatrics* **74,** 365–371 (2010).

70. Lin, Y. *et al.* Association study of genetic variants in eight genes/loci with type 2 diabetes in a Han Chinese population. *BMC Med. Genet.* **11,** 97 (2010).

71. Lu, Y.-L., Li, G.-L., Huang, H.-L., Zhong, J. & Dai, L.-C. Peroxisome proliferator-activated receptor-gamma 34C>G polymorphism and colorectal cancer risk: a meta-analysis. *World J. Gastroenterol.* **16,** 2170–2175 (2010).

72. Wen, J. *et al.* Investigation of type 2 diabetes risk alleles support CDKN2A/B, CDKAL1, and TCF7L2 as susceptibility genes in a Han Chinese cohort. *PloS One* **5,** e9153 (2010).

73. Xu, W. *et al.* PPARgamma polymorphisms and cancer risk: a meta-analysis involving 32,138 subjects. *Oncol Rep* **24,** 579–85 (2010).

74. Xu, M. *et al.* Combined effects of 19 common variations on type 2 diabetes in Chinese: results from two community-based studies. *PloS One* **5,** e14022 (2010).

75. Povel, C. M., Boer, J. M. A., Reiling, E. & Feskens, E. J. M. Genetic variants and the metabolic syndrome: a systematic review. *Obes. Rev.* **12,** 952–967 (2011).

76. Tang, H., Dong, X., Hassan, M., Abbruzzese, J. L. & Li, D. Body mass index and obesity- and diabetes-associated genotypes and risk for pancreatic cancer. *Cancer Epidemiol. Biomark. Prev.* **20,** 779–792 (2011).

77. Richardson, D. K. *et al.* Association between variants in the genes for adiponectin and its receptors with insulin resistance syndrome (IRS)-related phenotypes in Mexican Americans. *Diabetologia* **49,** 2317–2328 (2006).

78. Loos, R. J. F. *et al.* Adiponectin and adiponectin receptor gene variants in relation to resting metabolic rate, respiratory quotient, and adiposity-related phenotypes in the Quebec Family Study. *Am. J. Clin. Nutr.* **85,** 26–34 (2007).

79. Doecke, J. D. *et al.* Single nucleotide polymorphisms in obesity-related genes and the risk of esophageal cancers. *Cancer Epidemiol. Biomark. Prev.* **17,** 1007–1012 (2008).

80. Kaklamani, V. G. *et al.* Variants of the adiponectin and adiponectin receptor 1 genes and breast cancer risk. *Cancer Res.* **68,** 3178–3184 (2008).

81. Kaklamani, V. G. *et al.* Variants of the adiponectin (ADIPOQ) and adiponectin receptor 1 (ADIPOR1) genes and colorectal cancer risk. *JAMA* **300,** 1523–31 (2008).

82. Rasmussen-Torvik, L. J. *et al.* The association of SNPs in ADIPOQ, ADIPOR1, and ADIPOR2 with insulin sensitivity in a cohort of adolescents and their parents. *Hum. Genet.* **125,** 21–28 (2009).

83. Wang, Y. *et al.* Association study of the single nucleotide polymorphisms in adiponectin-associated genes with type 2 diabetes in Han Chinese. *J. Genet. Genomics* **36,** 417–423 (2009).

84. Beebe-Dimmer, J. L., Zuhlke, K. A., Ray, A. M., Lange, E. M. & Cooney, K. A. Genetic variation in adiponectin (ADIPOQ) and the type 1 receptor (ADIPOR1), obesity and prostate cancer in African Americans. *Prostate Cancer* **13,** 362–368 (2010).

85. Partida-Pérez, M. *et al.* Association of LEP and ADIPOQ common variants with colorectal cancer in Mexican patients. *Cancer Biomark. Sect. Dis. Markers* **7,** 117–121 (2010).

86. Al Khaldi, R. M., Al Mulla, F., Al Awadhi, S., Kapila, K. & Mojiminiyi, O. A. Associations of single nucleotide polymorphisms in the adiponectin gene with adiponectin levels and cardio-metabolic risk factors in patients with cancer. *Dis. Markers* **30,** 197–212 (2011).

87. He, B. *et al.* Effects of genetic variations in the adiponectin pathway genes on the risk of colorectal cancer in the Chinese population. *BMC Med Genet* **12,** 94 (2011).

88. Li, Y. *et al.* Association of adiponectin SNP+45 and SNP+276 with type 2 diabetes in Han Chinese populations: a meta-analysis of 26 case-control studies. *PloS One* **6,** e19686 (2011).

89. Yu, Z. *et al.* Genetic polymorphisms in adipokine genes and the risk of obesity: a systematic review and meta-analysis. *Obes.* **20,** 396–406 (2012).

90. Potapov, V. A. *et al.* Adiponectin and adiponectin receptor gene variants in relation to type 2 diabetes and insulin resistance-related phenotypes. *Rev. Diabet. Stud. RDS* **5,** 28–37 (2008).

91. Carvajal-Carmona, L. G. *et al.* Common variation at the adiponectin locus is not associated with colorectal cancer risk in the UK. *Hum Mol Genet* **18,** 1889–92 (2009).

92. Pechlivanis, S. *et al.* Genetic variation in adipokine genes and risk of colorectal cancer. *Eur. J. Endocrinol.* **160,** 933–940 (2009).

93. Schwarz, P. E. H. *et al.* Global meta-analysis of the C-11377G alteration in the ADIPOQ gene indicates the presence of population-specific effects: challenge for global health initiatives. *Pharmacogenomics J.* **9,** 42–48 (2009).

94. Gong, M., Long, J., Liu, Q. & Deng, H. C. Association of the ADIPOQ rs17360539 and rs266729 polymorphisms with type 2 diabetes: a meta-analysis. *Mol. Cell. Endocrinol.* **325,** 78–83 (2010).

95. Teras, L. R. *et al.* No association between polymorphisms in LEP, LEPR, ADIPOQ, ADIPOR1, or ADIPOR2 and postmenopausal breast cancer risk. *Cancer Epidemiol. Biomark. Prev.* **18,** 2553–2557 (2009).

96. Gornick, M. C., Rennert, G., Moreno, V. & Gruber, S. B. Adiponectin gene and risk of colorectal cancer. *Br. J. Cancer* **105,** 562–564 (2011).

97. Richards, J. B. *et al.* A genome-wide association study reveals variants in ARL15 that influence adiponectin levels. *PLoS Genet.* **5,** e1000768 (2009).

98. Damcott, C. M. *et al.* Genetic variation in adiponectin receptor 1 and adiponectin receptor 2 is associated with type 2 diabetes in the Old Order Amish. *Diabetes* **54,** 2245–2250 (2005).

99. Hara, K. *et al.* Absence of an association between the polymorphisms in the genes encoding adiponectin receptors and type 2 diabetes. *Diabetologia* **48,** 1307–1314 (2005).

100. Stefan, N. *et al.* Polymorphisms in the gene encoding adiponectin receptor 1 are associated with insulin resistance and high liver fat. *Diabetologia* **48,** 2282–2291 (2005).

101. Siitonen, N. *et al.* Association of sequence variations in the gene encoding adiponectin receptor 1 (ADIPOR1) with body size and insulin levels. The Finnish Diabetes Prevention Study. *Diabetologia* **49,** 1795–1805 (2006).

102. Vaxillaire, M. *et al.* Genetic analysis of ADIPOR1 and ADIPOR2 candidate polymorphisms for type 2 diabetes in the Caucasian population. *Diabetes* **55,** 856–861 (2006).

103. Collins, S. C. *et al.* Adiponectin receptor genes: mutation screening in syndromes of insulin resistance and association studies for type 2 diabetes and metabolic traits in UK populations. *Diabetologia* **50,** 555–562 (2007).

104. Crimmins, N. A. *et al.* Adiponectin receptor 1 variants associated with lower insulin resistance in African Americans. *Obes.* **15,** 1903–1907 (2007).

105. Qi, L., Doria, A., Giorgi, E. & Hu, F. B. Variations in adiponectin receptor genes and susceptibility to type 2 diabetes in women: a tagging-single nucleotide polymorphism haplotype analysis. *Diabetes* **56,** 1586–1591 (2007).

106. Cohen, S. S. *et al.* ADIPOQ, ADIPOR1, and ADIPOR2 polymorphisms in relation to serum adiponectin levels and BMI in black and white women. *Obes.* **19,** 2053–2062 (2011).

107. Yeh, E. *et al.* Association of polymorphisms at the ADIPOR1 regulatory region with type 2 diabetes and body mass index in a Brazilian population with European or African ancestry. *Braz. J. Med. Biol. Res. Rev.* **41,** 468–472 (2008).

108. Kim, J. T. *et al.* Polymorphisms of ADIPOR1 and ADIPOR2 are associated with phenotypes of type 2 diabetes in Koreans. *Clin. Endocrinol. (Oxf.)* **70,** 66–74 (2009).
